# Epidemiology and genetic diversity of *Taenia asiatica*: a systematic review

**DOI:** 10.1186/1756-3305-7-45

**Published:** 2014-01-22

**Authors:** Anita Ale, Bjorn Victor, Nicolas Praet, Sarah Gabriël, Niko Speybroeck, Pierre Dorny, Brecht Devleesschauwer

**Affiliations:** 1 National Zoonoses and Food Hygiene Research Center, Kathmandu, Nepal; 2 Department of Biomedical Sciences, Institute of Tropical Medicine, Antwerp, Belgium; 3 Institute of Health and Society (IRSS), Université catholique de Louvain, Brussels, Belgium; 4 Department of Virology, Parasitology and Immunology, Faculty of Veterinary Medicine, Ghent University, Merelbeke, Belgium

**Keywords:** Transmission, Geographical distribution, Genetic diversity, *Taenia asiatica*

## Abstract

*Taenia asiatica* has made a remarkable journey through the scientific literature of the past 50 years, starting with the paradoxical observation of high prevalences of *T. saginata*-like tapeworms in non-beef consuming populations, to the full description of its mitochondrial genome. Experimental studies conducted in the 1980s and 1990s have made it clear that the life cycle of *T. asiatica* is comparable to that of *T. saginata*, except for pigs being the preferential intermediate host and liver the preferential location of the cysts. Whether or not *T. asiatica* can cause human cysticercosis, as is the case for *Taenia solium*, remains unclear. Given the specific conditions needed to complete its life cycle, in particular the consumption of raw or poorly cooked pig liver, the transmission of *T. asiatica* shows an important ethno-geographical association. So far, *T. asiatica* has been identified in Taiwan, South Korea, Indonesia, the Philippines, Thailand, south-central China, Vietnam, Japan and Nepal. Especially this last observation indicates that its distribution is not restricted to South-East-Asia, as was thought so far. Indeed, the molecular tools developed over the last 20 years have made it increasingly possible to differentiate *T. asiatica* from other taeniids. Such tools also indicated that *T. asiatica* is related more closely to *T. saginata* than to *T. solium*, feeding the debate on its taxonomic status as a separate species versus a subspecies of *T. saginata*. Furthermore, the genetic diversity within *T. asiatica* appears to be very minimal, indicating that this parasite may be on the verge of extinction. However, recent studies have identified potential hybrids between *T. asiatica* and *T. saginata*, reopening the debate on the genetic diversity of *T. asiatica* and its status as a separate species.

## Background: the journey of a new tapeworm

The journey of *Taenia asiatica*, as documented by scientific literature, started in Taiwan in the late 1960s. Several authors reported on the paradox of observing a high prevalence of *Taenia saginata*-like tapeworms in the native aboriginal population living in mountainous areas of Taiwan, while these populations restrained from beef consumption (reviewed by [1,2]), and meat inspection for bovine cysticercosis had been negative for some time [3]. Dr Ping-Chin Fan, a Taiwanese parasitologist, conducted various studies on this Taiwan *Taenia* in the late 1980s and early 1990s (reviewed by [1,4-6]). Through observational and experimental studies, he and his team observed the morphology and researched the epidemiology of the Taiwan *Taenia*, which diverged from that of *T. saginata*. This led Fan to raise the possibility of the Taiwan *Taenia* being a new species [4].

Further experimental studies on *T. saginata*-like tapeworms from South Korea, Indonesia, Thailand, and the Philippines showed similar results, leading the authors to rename their Taiwan *Taenia* into Asian *Taenia*, denoting its more diverse geographical distribution [7-10].

In the early 1990s, a group of Korean parasitologists, led by Dr Keeseon Eom, performed various experimental infections using Korean specimens of the Asian *Taenia*. Their observation that the cysts of the Asian *Taenia* preferably develop in viscera of pigs, made them propose the name *Cysticercus viscerotropica*[11]. In 1993, they described the morphology of the Asian *Taenia*, and declared it as a new species, designated *Taenia asiatica*[12].

With the advent of molecular techniques, it became clear that the Asian *Taenia* is genetically much more related to *T. saginata* than it is to *T. solium*[13-17]. This led the earlier protagonists to declare the Asian *Taenia* as a strain or subspecies of *T. saginata*, designated *Taenia saginata taiwanensis*, or, in line with its geographical distribution, *Taenia saginata asiatica*[18].

However, this purely molecular view was soon contrasted to an epidemiological and public health perspective. Galan-Puchades and Mas-Coma opened the debate, and made the case for *T. asiatica* as a separate species [19]. The debate continued, with different phylogenetic studies considering the Asian *Taenia* either as *T. saginata asiatica*[20,21] or *T. asiatica*[22-27], depending on the research group involved. The identification of *T. saginata*/*T. asiatica* hybrids in China and Thailand [28-30], may reopen this debate, as reproductive isolation has historically been an important criterion for considering *T. asiatica* and *T. saginata* as distinct biological entities [24]. Although the current literature seems to favor *T. asiatica* at the species level, it is clear that the taxonomy of the Asian *Taenia* remains as complex and controversial as it was two decades ago [31].

The remainder of this systematic review will provide an update of our current understanding of the transmission, risk factors, geographical distribution and genetic diversity of *T. asiatica*. For a review on the history, taxonomy and morphology of *T. asiatica*, we refer to Eom [32].

## Search strategy

Evidence on the epidemiology and genetic diversity of *T. asiatica* was obtained through a systematic search of national and international peer-reviewed literature. Given the relatively limited number of papers on *T. asiatica*, a general search phrase was used consisting of the different synonyms of *T. asiatica*, i.e., *Taenia saginata asiatica*, Asian *Taenia* and Taiwan *Taenia*. Manuscript titles were retrieved through searching PubMed, Asia Journals OnLine (AsiaJOL), African Journals OnLine (AJOL), Latin American Journals OnLine (LAMJOL), WHO Global Health Library, and IndMED. The searches were performed on 20 September 2013.

In a second step, the retrieved titles were screened for eligibility by applying a set of inclusion/exclusion criteria to the titles and, if possible, to the abstracts and full texts. Papers were included if they provided authentic information on the transmission, geographic distribution and/or genetic diversity of *T. asiatica*. No restrictions were imposed on the publication year or on the language of the manuscript. Non-English manuscripts were translated through Google Translate (http://translate.google.be/).

In a third step, additional titles were retrieved by hand-searching the reference lists of the eligible documents initially retrieved. The same inclusion/exclusion criteria as for the initial titles were applied to these new titles. The backward reference searches were repeated until no more new information could be retrieved.

In a fourth and final step, a narrative synthesis of each paper was made, serving as the basis for the current qualitative review of the epidemiology and genetic diversity of *T. asiatica*.

**Figure 1:**
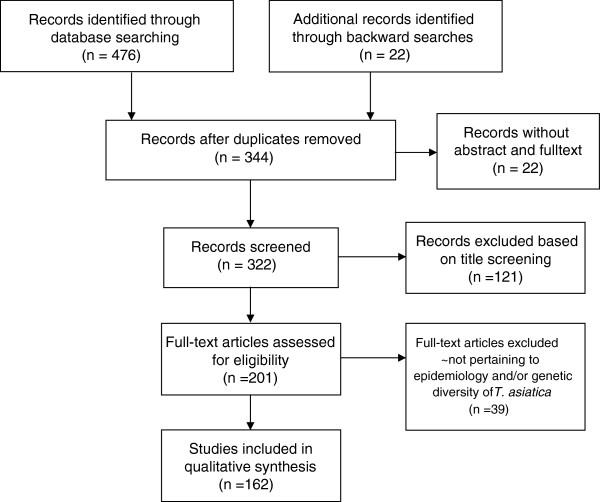
Flow diagram of applied search strategy.

Figure 1 shows a flow diagram of the applied search strategy. In total, 162 relevant unique records could be identified, of which 15 had to be translated from Chinese (n = 14) or Korean (n = 1). Of the 162 unique records, 49 provided information on the transmission and risk factors of *T. asiatica*; 92 on its geographical distribution; and 42 on its genetic diversity. Several studies provided information on multiple aspects covered by this review.

## Review

### Transmission and risk factors

The transmission and infectivity potential of *T. asiatica* has been extensively studied in the 1980s and 1990s through experimental studies [1,4,11,33]. Humans act as definite host, while pigs appear to act as natural intermediate hosts. However, other animals, such as cattle, goats and certain monkey species were also found to become infected [1,8-11,34]. Experimental infections with the eggs of Asian *Taenia* in cats, dogs, rabbits, rodents and baboons (*Papio hamadryas*), on the other hand, were not successful [1,35].

The life cycle of *T. asiatica* appears to be rather short compared to other human *Taenia* species. Approximately four weeks after ingestion of eggs, mature cysts become visible in the intermediate host [1,11]. *T. asiatica* cysts are mainly found in the liver of intermediate hosts, and more so in the parenchyma than on the surface. Other viscera, such as lungs, omentum, serosa and mesentery, may also harbor cysts [11,34,36]. These extrahepatic cysts are believed to have migrated from the liver surface [34]. Two to four months after ingestion of viable cysts by a human host, motile tapeworm segments may be excreted with the stool [33,37,38]. The number of proglottids released per day may vary from 0 to 35 [37,38]. Cases have been reported of carriers passing proglottids for more than 30 years [39,40], although it cannot be ascertained if this was due to the same tapeworm or due to reinfection.

Comparatively, the life cycle of *T. asiatica* closely follows that of *T. saginata*, with the exception of the natural intermediate host (pigs versus cattle), and the location in the intermediate host (liver versus muscle). A matter of debate remains whether *T. asiatica* may also infect humans as intermediate host, as is the case for the other human taeniid, *T. solium*. Experimental infections with *T. asiatica* eggs in primates remain inconclusive, as Fall et al*.*[35] were not able to infect baboons, but other researchers did report successful infections in *Macaca cyclopis* and other unspecified monkey species [1,34]. Galan-Puchades & Fuentes [41,42] argue that, if *T. asiatica* were to cause cysticercosis, the location of the cysts would most likely be the liver, as in other intermediate hosts. Hepatic cysticercosis, if existing, would most probably not give rise to clinical symptoms, given the small size of the cysts. On the other hand, from a diagnostic point of view, cross-reactions in tests for the detection of other *Taenia* spp. would be very plausible, as cross-immunity and *in vitro* cross-reactions have been observed at various times [43-48].

The life cycle of *T. asiatica* indicates that the main transmission risk factors are raw pork viscera consumption (to infect the human host), and open defecation (to infect the intermediate host) [32]. As the consumption of (raw) pork viscera is generally less popular than that of (raw) pork meat, *T. asiatica* cannot efficiently spread within and between countries and acquire a true cosmopolitan status. However, social, cultural, and religious practices have preserved raw pork liver consumption in certain population groups, leading to high prevalences in these specific population groups, who often live in specific geographical areas. Indeed, observations from various countries demonstrate that transmission of *T. asiatica* is clearly ethnically and geographically associated. Table 1 gives an overview of the different ethno-geographical foci where *T. asiatica* has been studied. Remarkably, most of these foci were islands, which possibly facilitated the preservation of *T. asiatica* in these foci.

**Table 1:** **Ethno-geographical foci of *Taenia asiatica *** **transmission**

**Country**	**Region**	**People**	**Food habits**	**References**
Taiwan	Mountainous areas of northern and eastern Taiwan	Bunun, Atayal	Habit of eating viscera, especially liver and blood, of fresh-killed animals, including wild boar, but excluding cattle	Fan 1988 [1]; Chung *et al.* 1990 [39]; Fan *et al.* 1990 [49]; Fan *et al.* 1992a [40]; Ooi *et al.* 2013 [2]
Taiwan	Orchid island, Lanyu Township, Taitung County, southeastern Taiwan	Tao (originally known as Yami)	Habit of eating viscera, especially liver and blood, of fresh-killed animals, including wild boar, but excluding cattle	Fan *et al.* 1992b [50]; Eom *et al.* 2009 [51]; Ooi *et al.* 2013 [2]
Indonesia	Ambarita village, Samosir Island, northern Sumatra	Batak (Christian)	*“Sang-sang”*: traditional dish with minced pork, viscera and blood; during preparation, uncooked meat and viscera are sometimes eaten	Fan *et al.* 1989 [7]; Fan *et al.* 1992c [52]; Suroso *et al.* 2006 [53]; Wandra *et al.* 2006 [54]
South Korea	Jeju Island (Jeju-do) and mainland South Korea	*Not minority people as seen in other countries*	Habit of eating liver and other viscera of pigs at *“Churyum”*, a common rural practice of slaughtering pigs at home during weddings, funerals and other special occasions	Fan *et al.* 1992b [50]; Eom *et al.* 1992 [11]; Eom & Rim 2001 [55]; Galan-Puchades & Fuentes 2001 [56]
China	Luzhai County, Guangxi Zhuang Autonomous Region, southern China	Zhuang	Habit of eating raw pork and pig liver, unseasoned or mixed with sour sauce and salted garlic; consumption of raw beef is uncommon	Eom *et al.* 2002 [57]; Eom *et al.* 2009 [51]
China	Yajiang (Nyagqu) County, Garzê Tibetan Autonomous Prefecture, Sichuan Province	Kham Tibetans	Habit of eating raw pork and beef	Li *et al.* 2006 [58]; Li *et al.* 2013 [59]
Thailand	Thong Pha Phum District, Kanchanaburi Province, west-central Thailand, Thai-Myanmar border	Karen	Habit of eating raw or under cooked beef, pork, or pig viscera and fresh blood	Anantaphruti *et al.* 2007 [60]; Anantaphruti *et al.* 2010 [61]; Anantaphruti 2013 [62]
Japan	Kanto region, central Honshu	*Not minority people as seen in other countries*	Serving of pig liver *“sashimi”* (raw slices) at *“yakitori”* or *“yakiniku”* restaurants	Eom *et al.* 2009 [51]; Michelet & Dauga 2012 [63]; Yamasaki 2013 [64]
Nepal	Morang & Sunsari district, southeastern Nepal	Dum	Frequent pork consumption; habit of eating undercooked meat and viscera of home raised *Hurra* piglets during certain religious and social festivities	Devleesschauwer *et al.* 2012 [65]

Earlier studies reported an increasing prevalence with age [1,50,52], while others reported a predominance in males [62]. However, these findings can probably be attributed to differential consumption patterns.

### Geographical distribution

**Figure 2:**
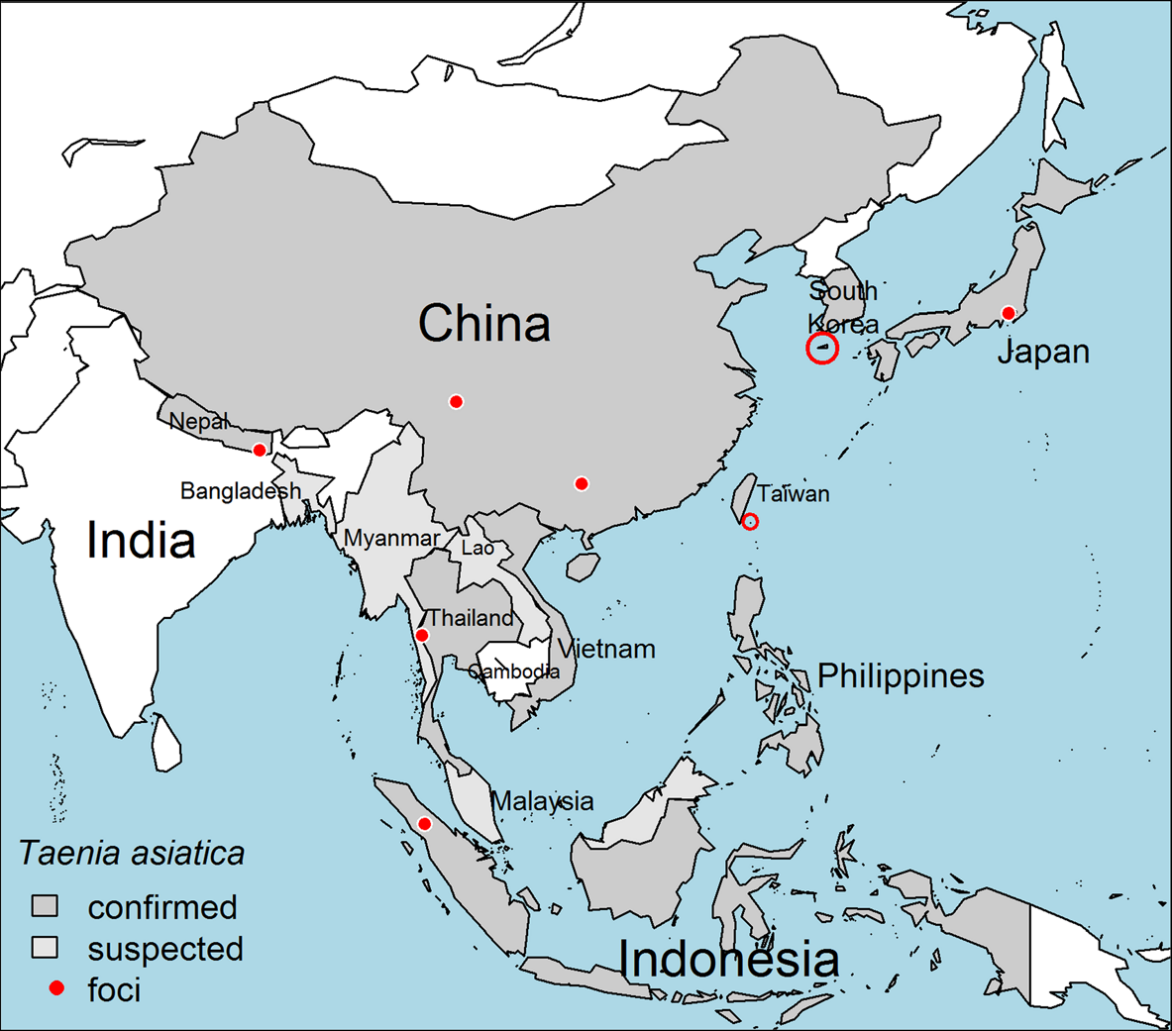
**Country-level geographical distribution of *Taenia asiatica*.** In China, *T. asiatica* has so far only been identified in the south-central provinces Yunnan, Guangxi, Guizhou and Sichuan. The ethno-geographical foci refer to, from west to east: Morang & Itahari district (Nepal); Thong Pha Phum district (Kanchanaburi province; Thailand); Samosir Island (North Sumatra, Indonesia); Yajiang (Nyagqu) County (Garzê Tibetan Autonomous Prefecture, Sichuan Province, China); Luzhai County (Guangxi Zhuang Autonomous Region, China); Orchid island (Lanyu Township, Taitung County, Taiwan); Jeju Island (Jeju-do, South Korea); and Kanto region (Honshu, Japan).

The current state of knowledge on the geographical distribution of *T. asiatica* is visualized in Figure 2. Most knowledge originates from the analysis of individual specimens and limited case series. Observational studies on *T. asiatica* remain largely lacking, mainly due to the difficulty in identifying tapeworm carriers and the subsequent collection and identification of tapeworm specimens. As a result, the true prevalence of *T. asiatica* taeniosis remains unknown.

To date, most observations originate from the countries where *T. asiatica* was initially studied, i.e., Taiwan and South Korea. In fact, high taeniosis prevalences have been reported from these countries since the beginning of the 20th century, although it is unclear to what extent these cases were due to *T. asiatica*. The first report from Taiwan is ascribed to Oi (1915; cited by [2]), while the first report from South Korea is ascribed to Matsumoto (1915; cited by [55]). Zarlenga *et al.*[13] were the first to confirm *T. asiatica* specimens at the molecular level in these countries, and several studies would follow (e.g., [15,29,57,66-69]). Interestingly, Jeon *et al.*[70] found that 51 out of 68 museum specimens, preserved since 1935–2005 and originating from different South Korean provinces, were in fact *T. asiatica*, whereas they were initially labeled as *T. saginata*. Current knowledge on taeniosis and *T. asiatica* in Taiwan and South Korea is reviewed by, respectively, [2], and [55,71].

Fan *et al*. identified in their original studies the presence of *T. asiatica* in Indonesia, Thailand and the Philippines based on morphological characteristics [7,9,10]. These findings were later confirmed by molecular characterization of individual specimens originating from these countries. *T. asiatica* has so far mainly been identified on Sumatra [29,72]. During 2003–2005, an epidemiological survey on 240 local people identified six *T. asiatica* tapeworm carriers (2.5%), using mitochondrial DNA analysis [53,54]. The presence of *T. asiatica* on Bali, on the other hand, remains controversial. No human *T. asiatica* taeniosis cases have been confirmed [54,73,74], but in 1998, 146 of 638 pigs (22.9%) at a slaughter-house in Bali were found to have cysts in the liver [31], and more recently, one cyst originating from Bali was identified as *T. asiatica* by multiplex Polymerase Chain Reaction (PCR) [67]. The current knowledge on taeniosis and cysticercosis in Indonesia has recently also been reviewed by Wandra *et al.*[75]. In Thailand, different *T. asiatica* cases, including one triple infection and one co-infection with *T. solium*, have been identified in the Myanmar-bordering Kanchanaburi province [29,60,61]. The current status of taeniasis in Thailand is further reviewed by Anantaphruti [62]. In the Philippines, finally, different authors identified individual *T. asiatica* specimens [29,68,69], but no epidemiological studies appear to have been performed.

McManus & Bowles [15] report the first confirmation of *T. asiatica* from mainland China, but without providing details on the exact origin of the examined specimen. Further research identified *T. asiatica* cases in four south-central provinces of China, i.e., Yunnan, Guangxi, Guizhou and Sichuan. Zhang *et al.*[36] were the first to identify *T. asiatica* in Yunnan province, based on morphological characteristics of adults and cysts. To date, specimens have been molecularly confirmed from three Yunnan counties, i.e., Lanpin, Dali and Baoshan [20,76-78]. Eom *et al.*[57] identified six cases from the Zhuang minority in Guangxi province. Other specimens have been confirmed from Luzhai and Binyang county in Guangxi [68,79]. Furthermore, cases have been confirmed from Duyun and Congjiang county in Guizhou province [20,76,80,81], and from Yajiang and Danba county in Sichuan province [30,58,82].

More recently, it has become clear that the geographical distribution of *T. asiatica* is more widespread than originally thought, as cases have been confirmed in Vietnam, Japan and Nepal. In 2003, a case report was published on a patient from Ha Tay province, Vietnam, suffering from taeniosis [83]. DNA analysis revealed that the causative species was *T. asiatica*. Somers *et al.*[84] confirmed 36 out of 65 human tapeworm cases in North Vietnam as *T. asiatica*. In 2010, the *Infectious Agents Surveillance Report* of the Japanese National Institute of Infectious Diseases dedicated a section to the detection of several *T. asiatica* cases in Kanto region (reviewed by [64,85]). These were the first and so far only case reports from Japan, although two museum samples had earlier been molecularly characterized as *T. asiatica*[51,72]. In Nepal, several *T. asiatica* tapeworm carriers were identified among the Dum, an indigenous community living along the Nepal-India border [65]. So far, this is the most western location where *T. asiatica* has been found.

For other South-Asian and South-East-Asian countries, i.e., Bangladesh, Myanmar, Malaysia, Lao PDR, and Cambodia, there is considerable uncertainty regarding the presence of *T. asiatica*. Amin *et al.*[86] reported a case of a symptomatic tapeworm carrier from rural Bangladesh. As the patient reportedly had not consumed undercooked beef, but possibly undercooked pork, the case was ascribed to *T. asiatica*. Nevertheless, as no morphological or molecular identification was performed, this diagnosis remains questionable. No cases have been confirmed from Myanmar, but Anantaphruti [62] identified *T. asiatica* in a Karen immigrant who recently moved from Myanmar to Kanchanaburi province, Thailand, and was passing segments in stool before migration. McManus & Bowles [15] identified one specimen from Malaysia as *T. asiatica*, and Conlan *et al.*[74] mentioned the finding of *T. asiatica* in one of 590 pig livers sampled in Lao PDR, but further evidence for these countries remains lacking. In Cambodia, finally, the only identification study conducted so far could not identify *T. asiatica* among 21 tapeworm carriers [87].

### Genetic diversity

Since the 1990s, various researchers have developed and applied molecular methods for distinguishing *T. asiatica* from other *Taenia* spp., and, subsequently, for studying the relationship of *T. asiatica* with other *Taenia* spp., in particular *T. saginata*, and for examining the genetic variability within *T. asiatica*. The availability of the complete mitochondrial genome of *T. asiatica*[27], and the access to published sequences through GenBank, has greatly contributed to the understanding of the genetic diversity of *T. asiatica*.

#### Molecular tools for *Taenia asiatica* identification

Different tools have been developed to differentiate *T. asiatica* from other *Taenia* species based on the genetic information available in proglottids, cysts and eggs. Table 2 gives an overview of the different molecular tools and markers developed so far. To date, the most common method for molecular identification of *Taenia* tapeworms has been PCR coupled with nucleotide sequencing of the amplified PCR product. Different mitochondrial (e.g., cytochrome c oxidase subunit I [*COX-1*], cytochrome b [*COB*], NADH dehydrogenase subunit I [*NAD-1*], and 12 s ribosomal RNA) and nuclear (e.g., ribosomal RNA (i.e., 18S rRNA, 5.8S rRNA, internal transcribed spacer 2 [*ITS-2*], and 28S rRNA), elongation factor-1-alpha [*ef1*], and ezrin/radixin/moesin-like protein [*elp*]) genes have been used as markers in such analyses (Table 2). By aligning the obtained sequences with published ones, the identity of the specimens can be determined [21]. Other assays for the differential diagnosis of *Taenia* tapeworms include PCR [13,69,93], PCR coupled with Restriction Fragment Length Polymorphism analysis (PCR-RFLP; [13,15,84,93,94]), Random Amplified Polymorphic DNA analysis (RAPD; [20,57]), Base Excision Sequence Scanning Thymine-base analysis (BESS T-base; [66]), multiplex PCR [67,68,93], and Loop-Mediated Isothermal Amplification (LAMP; [28]).

**Table 2:** **Molecular tools for *T. asiatica *** **identification**

**Method marker**		**Restriction enzymes**	**References**
*Nucleotide sequencing*			
	mt-*COX-1*	–	McManus & Bowles 1994 [15]; Bowles & McManus 1994 [16]; McManus 1997 [17]; Gasser *et al.* 1999 [88]; Wang & Bao 2003 [76]; Yamasaki *et al.* 2005 [89]; Jeon *et al.* 2008 [70]; Okamoto *et al.* 2010 [29]; Jeon *et al.* 2011a [87]; Jeon *et al.* 2011b [72]; Yang *et al.* 2012 [79]
	28S rDNA	–	McManus & Bowles 1994 [15]; Bowles & McManus 1994 [16]; McManus 1997 [17]
	mt-*NAD-1*	–	Gasser *et al.* 1999 [88]
	5.8S rDNA/*ITS-2*/28S rDNA	–	Eom *et al.* 2002 [57]; Jeon *et al.* 2008 [70]
	mt-*COB*	–	Le *et al.* 2003 [90]; Yamasaki *et al.* 2005 [89]
	*HDP2*	–	Gonzalez *et al.* 2010 [69]
	*ef1*	–	Okamoto *et al.* 2010 [29]
	*elp*	–	Okamoto *et al.* 2010 [29]
	*18 kDa/HP6*	–	Gonzalez *et al.* 2011 [91]
	mt-12S rDNA	–	Liu & Yang 2011 [77]
	18S rDNA	–	Yan *et al.* 2013 [78]
*PCR*			
	Ribosomal DNA	–	Zarlenga *et al.* 1991 [13]; Zarlenga 1991 [14]; Morakote *et al.* 2000 [92]
	*HDP2*	–	Gonzalez *et al.* 2004 [93]
	*HDP2*	–	Gonzalez *et al.* 2010 [69]
*PCR-RFLP*			
	Ribosomal DNA	*Bam*HI	Zarlenga *et al.* 1991 [13]; Zarlenga 1991 [14]
	mt-*COX-1*	*Msp*I	McManus & Bowles 1994 [15]; Bowles & McManus 1994 [16]; McManus 1997 [17]
	*rDNA ITS-1*	*Msp*I*, Cfo*I*, Rsa*I	McManus & Bowles 1994 [15]; Bowles & McManus 1994 [16]; McManus 1997 [17]
	Mitochondrial DNA	*Eco*RI, *Hind*III, *Bg*III, *Xba*I, *Bam*HI, *Pvu*II, *Ava*I, *Hae*III, *Hinc*II, *Eco*RV, *Hpa*II	Zarlenga & George 1995 [94]
	*HDP2*	*Bcl*I or *Bgl*I	Gonzalez *et al.* 2004 [93]
	12S rDNA	*Dde*I, *Hinf*I	Somers *et al.* 2007 [84]; Devleesschauwer *et al.* 2012 [65]
*RAPD analysis*			
	3 random primers: OPA-03, OPA-08, OPA-20	–	Eom *et al.* 2002 [57]
	13 random primers: OPA-03, OPA-08, OPA-20, L-01, L-02, L-05, L-06, L-08, L-09, L-12, L-15, L-18, L-19	–	Zhang *et al.* 2006 [20]
*BESS T-base analysis*			
	mt-*COX-1*	–	Yamasaki *et al.* 2002 [66]
	mt-*COB*	–	Yamasaki *et al.* 2002 [66]
*Multiplex PCR*			
	mt-*COX-1*	–	Yamasaki *et al.* 2004 [67]
	*HDP2*	–	Gonzalez *et al.* 2004 [93]
	mt-Valine tRNA, mt-*NAD-2,* mt-*NAD-1*	–	Jeon *et al.* 2009 [68]; Jeon *et al.* 2011a [87]
*LAMP*			
	mt-*COX-1*	–	Nkouawa *et al.* 2009 [28]; Nkouawa *et al.* 2012 [82]
	cathepsin L-like cysteine peptidase [*clp*]	*Hinf*I	Nkouawa *et al.* 2009 [28]

#### Genetic relatedness of *Taenia asiatica*, *Taenia saginata* and *Taenia solium*

With the availability of the mitochondrial genome of the different human taeniids, their genetic relatedness has become clearer [25-27]. The mitochondrial genomes of *T. asiatica*, *T. saginata* and *T. solium* measure 13,703 bp, 13,670 bp, and 13,709 bp, respectively. They consist of 36 genes, i.e., 12 protein-coding genes, 22 tRNA genes, and 2 rRNA genes. There is an overall nucleotide difference of 4.6% between *T. asiatica* and *T. saginata*, and of 11% between *T. saginata* and *T. solium*. The nucleotide difference in the *COX-1* gene is 4.6% for *T. asiatica*/*T. saginata*, 12.3% for *T. saginata*/*T. solium*, and 12.0% for *T. asiatica*/*T. solium*. These results thus indicate that *T. asiatica* is more closely related to *T. saginata* than it is to *T. solium*.

Similar conclusions are obtained from sequence comparisons of nuclear genes. The 18 kDa/HP6 protein-encoding gene shows a similarity of 95.5% between *T. asiatica* and *T. saginata*, but a similarity of merely 61.5% between *T. saginata* and *T. solium*[91]. The 18S rRNA genes of *T. asiatica* and *T. saginata* appeared to be 99.2% identical [78].

Studies on the diversification times of the different *Taenia* species indicate that *T. asiatica* fully diverged from a common *T. saginata/asiatica* ancestor in the late Pleistocene, ~40,000 years ago. This diversification might have co-occurred with the arrival of *Homo sapiens* in Asia, and the introduction of new wild boar populations and/or new breeding and husbandry practices in this region [63,95]. Again, these results indicate a closer relatedness of *T. asiatica* with *T. saginata* than with *T. solium*.

#### Genetic variability within *Taenia asiatica*

Several studies have indicated little or no genetic variation within *T. asiatica*. The nucleotide diversity found in the mitochondrial *COX-1* gene of specimens from different regions ranged between 0 and 0.2% [16,26,67]. *T. saginata*, on the other hand, appears to show an important genetic polymorphism, with mitochondrial *COX-1* gene diversity ranging from 0.2 to 0.8% [26]. In an analysis of *COX-1* gene sequences of 30 *T. asiatica* specimens from seven countries, only two haplotypes could be identified, with the major haplotype comprising 29 out of 30 samples. *T. saginata*, on the other hand, was found to be much more polymorphic, with eight haplotypes and a ten times higher haplotype diversity (0.70 versus 0.07; [96,97]).

The limited amount of genetic variation within *T. asiatica* has also been observed for other genes. Indeed, sequence divergences of 0.1 to 2.1% were found in *T. asiatica* HDP2 fragments [69], while no divergence was found in the 18 kDa gene sequence [91].

In contrast with this low genetic diversity is the recent identification of potential hybrids between *T. asiatica* and *T. saginata*. Nkouawa *et al.*[28] identified two specimens, one from China and one from Thailand, that were identified as *T. saginata* based on the mitochondrial *COX-1* gene, but as *T. asiatica* based on the nuclear cathepsin L-like cysteine peptidase gene. It is therefore possible that these specimens were hybrid parasites with *T. saginata* mitochondrial DNA and *T. asiatica* nuclear DNA. Likewise, a discrepancy between the mitochondrial and nuclear DNA was found in two tapeworm specimens from Thailand [29]. While the specimens had sequences typical of the *T. saginata* COX-1 gene, both were homozygous for *T. asiatica* typical alleles of the *elp* gene, and one was additionally homozygous for *T. asiatica* typical alleles of the *ef1* gene. A similar mitochondrial/nuclear discordance was observed in China, where one tapeworm with *T. asiatica* mitochondrial DNA was heterozygous for *T. saginata* typical alleles of the *elp* and *ef1* genes, while a second tapeworm, with *T. saginata* mitochondrial DNA, was heterozygous for *T. asiatica* typical alleles of the *ef1* gene [30].

## Conclusions

In the past 50 years, *T. asiatica* has made a remarkable journey through the scientific literature, from epidemiological paradox to mitochondrial genome. The studies performed in the 1980s and 1990s have provided us with the basic understanding of its transmission and risk factors. Nevertheless, several critical questions remain unanswered. As studies on the *T. asiatica* transmission risk factors so far mainly focused on pork liver consumption, other important factors, such as pig husbandry and slaughter practices, remain unaddressed. Furthermore, the potential of *T. asiatica* to cause human cysticercosis needs to be clarified. If this would be the case, then *T. asiatica* could be an important cause of cross reactions in serological assays, but, more importantly, of human illness, making this indeed the most neglected of all neglected tropical diseases [98]. Finally, more needs to be done to understand if and how *T. asiatica* can protect against *T. solium*, through density-dependent and/or immune-mediated processes [99]. Indeed, as humans may become infected with three different *Taenia* species, i.e., *T. asiatica*, *T. saginata* and *T. solium*, the presence of one tapeworm species might physically reduce the likelihood of a second species to develop. Likewise, as pigs can become infected with three *Taenia* species, i.e., *T. asiatica*, *T. solium* and *T. hydatigena*, cross-immunity may interfere with the establishment of cysts of other species. If such interspecific competition could be proven to play an important role in moderating *T. solium* transmission, *T. asiatica* would indeed be a parasite to treasure and to save from extinction [100].

With increasing access to molecular tools, the geographical distribution of *T. asiatica* has been shown to be much more widespread than initially thought. Indeed, the recent confirmation of *T. asiatica* in Nepal shows that its distribution is not restricted to South-East-Asia, as was thought so far. Further studies are warranted to identify the true spread of this parasite.

Over the last 20 years, genetic studies have provided important insights into the past, present and future of *T. asiatica*. The limited genetic diversity of *T. asiatica* specimens may be a signal that *T. asiatica* is an endangered species [96,97]. Indeed, as its transmission is rooted in often ancient sociocultural habits, the gradual disappearance of these habits along with global development and globalization, may lead to the spontaneous extinction of *T. asiatica*. Such a scenario is certainly not unrealistic, as it happened for *T. solium* in Europe [101] and observations from Indonesia and South Korea indicate that with changing habits, taeniosis prevalences decrease [102]. Finally, the significance of putative hybrids needs to be clarified, and more studies are needed to ascertain whether or not hybridization between *T. asiatica* and other species still occurs today.

## Abbreviations

BESS T-base: Base excision sequence scanning thymine-base; COX-1: Cytochrome c oxidase subunit I; COB: Cytochrome b; ef1: Elongation factor-1-alpha; elp: ezrin/radixin/moesin-like protein; ITS-2: Internal transcribed spacer 2; LAMP: Loop-mediated isothermal amplification; NAD-1: NADH dehydrogenase subunit I; PCR: Polymerase chain reaction; RAPD: Random amplified polymorphic DNA; RFLP: Restriction fragment length polymorphism; rRNA: ribosomal ribonucleic acid.

## Competing interests

The authors declare that they have no competing interests.

## Authors’ contributions

AA and BD performed the systematic literature review. AA and BD conceived and wrote the review with assistance from BV, NP, SG, NS, and PD. All authors read and approved the final manuscript.

## References

[B1] Fan PC Taiwan *Taenia* and taeniasis Parasitol Today 1988 4 86 88 10.1016/0169-4758(88)90204-9 15463051

[B2] Ooi HK Ho CM Chung WC Historical overview of *Taenia asiatica* in Taiwan Korean J Parasitol 2013 51 31 36 10.3347/kjp.2013.51.1.31 23467308 PMC3587746

[B3] Rhoads ML Murrell KD Cross JH Fan PC The serological response of pigs experimentally infected with a species of *Taenia* from Taiwan Vet Parasitol 1989 30 279 285 10.1016/0304-4017(89)90097-6 2728318

[B4] Fan PC Asian *Taenia saginata*: species or strain? Southeast Asian J Trop Med Public Health 1991 22 Suppl 245 250 1822898

[B5] Fan PC Review of taeniasis in Asia Zhonghua Min Guo Wei Sheng Wu Ji Mian Yi Xue Za Zhi 1995 28 79 94 9774987

[B6] Fan PC Chung WC *Taenia saginata asiatica*: epidemiology, infection, immunological and molecular studies J Microbiol Immunol Infect 1998 31 84 89 10596984

[B7] Fan PC Lin CY Kosman ML Kosin E Experimental infection of Indonesia *Taenia* (Samosir strain) in domestic animals Int J Parasitol 1989 19 809 812 10.1016/0020-7519(89)90070-2 2592148

[B8] Fan PC Lin CY Wu CC Chung WC Soh CT Experimental studies of Korea *Taenia* (Cheju strain) infection in domestic animals Ann Trop Med Parasitol 1989 83 395 403 2604477 10.1080/00034983.1989.11812363

[B9] Fan PC Chung WC Lin CY Wu CC Experimental infection of Thailand *Taenia* (Chiengmai strain) in domestic animals Int J Parasitol 1990 20 121 123 10.1016/0020-7519(90)90183-N 2312220

[B10] Fan PC Lin CY Chung WC Experimental infection of Philippine *Taenia* in domestic animals Int J Parasitol 1992 22 235 238 10.1016/0020-7519(92)90107-V 1587689

[B11] Eom KS Rim HJ Geerts S Experimental infection of pigs and cattle with eggs of Asian *Taenia saginata* with special reference to its extrahepatic viscerotropism Kisaengchunghak Chapchi 1992 30 269 275 1297417 10.3347/kjp.1992.30.4.269

[B12] Eom KS Rim HJ Morphologic descriptions of *Taenia asiatica* sp. n Korean J Parasitol 1993 31 1 6 10.3347/kjp.1993.31.1.1 8512894

[B13] Zarlenga DS McManus DP Fan PC Cross JH Characterization and detection of a newly described Asian taeniid using cloned ribosomal DNA fragments and sequence amplification by the polymerase chain reaction Exp Parasitol 1991 72 174 183 10.1016/0014-4894(91)90135-J 1672653

[B14] Zarlenga DS The differentiation of a newly described Asian taeniid from *Taenia saginata* using enzymatically amplified non-transcribed ribosomal DNA repeat sequences Southeast Asian J Trop Med Public Health 1991 22 Suppl 251 255 1822899

[B15] McManus DP Bowles J Asian (Taiwan) *Taenia*: species or strain? Parasitol Today 1994 10 273 275 10.1016/0169-4758(94)90145-7 15275445

[B16] Bowles J McManus DP Genetic characterization of the Asian *Taenia*, a newly described taeniid cestode of humans Am J Trop Med Hyg 1994 50 33 44 7905720

[B17] McManus DP Molecular genetic variation in *Echinococcus* and *Taenia*: an update Southeast Asian J Trop Med Public Health 1997 28 Suppl 1 110 116 9656360

[B18] Fan PC Lin CY Chen CC Chung WC Morphological description of *Taenia saginata asiatica* (Cyclophyllidea: Taeniidae) from man in Asia J Helminthol 1995 69 299 303 10.1017/S0022149X00014863 8583124

[B19] Galan-Puchades MT Mas-Coma S Considering *Taenia asiatica* at species level Parasitol Today 1996 12 123 author reply 123 15275245 10.1016/0169-4758(96)80674-0

[B20] Zhang K Yang M Bao HE The random amplified polymorphic DNA identification of 9 *Taenia saginata* isolates from four provinces Zhongguo Ji Sheng Chong Xue Yu Ji Sheng Chong Bing Za Zhi 2006 24 420 424 17366970

[B21] McManus DP Molecular discrimination of taeniid cestodes Parasitol Int 2006 55 Suppl S31 37 16337179 10.1016/j.parint.2005.11.004

[B22] De Queiroz A Alkire NL The phylogenetic placement of *Taenia* cestodes that parasitize humans J Parasitol 1998 84 379 10.2307/3284501 9576516

[B23] Hoberg EP Jones A Rausch RL Eom KS Gardner SL A phylogenetic hypothesis for species of the genus *Taenia* (Eucestoda: Taeniidae) J Parasitol 2000 86 89 98 10701570 10.1645/0022-3395(2000)086[0089:APHFSO]2.0.CO;2

[B24] Hoberg EP Phylogeny of *Taenia*: Species definitions and origins of human parasites Parasitol Int 2006 55 Suppl S23 30 16371252 10.1016/j.parint.2005.11.049

[B25] Jeon HK Kim KH Eom KS Complete sequence of the mitochondrial genome of *Taenia saginata*: comparison with *T. solium* and *T. asiatica* Parasitol Int 2007 56 243 246 10.1016/j.parint.2007.04.001 17499016

[B26] Jeon HK Eom KS *Taenia asiatica* and *Taenia saginata*: genetic divergence estimated from their mitochondrial genomes Exp Parasitol 2006 113 58 61 10.1016/j.exppara.2005.11.018 16546174

[B27] Jeon HK Lee KH Kim KH Hwang UW Eom KS Complete sequence and structure of the mitochondrial genome of the human tapeworm, *Taenia asiatica* (Platyhelminthes; Cestoda) Parasitology 2005 130 717 726 10.1017/S0031182004007164 15977909

[B28] Nkouawa A Sako Y Nakao M Nakaya K Ito A Loop-mediated isothermal amplification method for differentiation and rapid detection of *Taenia* species J Clin Microbiol 2009 47 168 174 10.1128/JCM.01573-08 19005142 PMC2620829

[B29] Okamoto M Nakao M Blair D Anantaphruti MT Waikagul J Ito A Evidence of hybridization between *Taenia saginata* and *Taenia asiatica* Parasitol Int 2010 59 70 74 10.1016/j.parint.2009.10.007 19874910

[B30] Yamane K Suzuki Y Tachi E Li T Chen X Nakao M Nkouawa A Yanagida T Sako Y Ito A Recent hybridization between *Taenia asiatica* and *Taenia saginata* Parasitol Int 2012 61 351 355 10.1016/j.parint.2012.01.005 22301089

[B31] Simanjuntak G Margono S Okamoto M Ito A Taeniasis/cysticercosis in Indonesia as an emerging disease Parasitol Today 1997 13 321 323 10.1016/S0169-4758(97)01104-6

[B32] Eom KS What is Asian *Taenia*? Parasitol Int 2006 55 Suppl S137 141 16387528 10.1016/j.parint.2005.11.022

[B33] Eom KS Rim HJ Experimental human infection with Asian *Taenia saginata* metacestodes obtained from naturally infected Korean domestic pigs Kisaengchunghak Chapchi 1992 30 21 24 1576110 10.3347/kjp.1992.30.1.21

[B34] Chung WC Lin CY Fan PC Ectopic locations of *Taenia saginata asiatica* cysticerci in the abdominal cavity of domestic pig and monkey J Parasitol 1996 82 1032 1034 10.2307/3284219 8973419

[B35] Fall EH Geerts S Kumar V Vervoort T De Deken R Eom KS Failure of experimental infection of baboons (*Papio hamadryas*) with the eggs of Asian *Taenia* J Helminthol 1995 69 367 368 10.1017/S0022149X00014978 8583131

[B36] Zhang L Tao H Zhang B Wang H Wang Y Li Z Yang J Yang B Li Y Pang Y First discovery of *Taenia saginata asiatica* infection in Yunnan province Zhongguo Ji Sheng Chong Xue Yu Ji Sheng Chong Bing Za Zhi 1999 17 95 96 12563790

[B37] Chao D Wong MM Fan PC Experimental infection in a human subject by a possibly undescribed species of *Taenia* in Taiwan J Helminthol 1988 62 235 242 10.1017/S0022149X00011585 3192916

[B38] Chang SL Ooi HK Nonaka N Kamiya M Oku Y Development of *Taenia asiatica* cysticerci to infective stage and adult stage in Mongolian gerbils J Helminthol 2006 80 219 223 16923263

[B39] Chung WC Fan PC Lin CY Wu CC Studies of taeniasis in Taiwan. XII. Prevalence of taeniasis among Atayal aborigines in Wufeng District, Hsinchu County, northwest Taiwan Gaoxiong Yi Xue Ke Xue Za Zhi 1990 6 66 72 2352317

[B40] Fan PC Chung WC Lin CY Chan CH Clinical manifestations of taeniasis in Taiwan aborigines J Helminthol 1992 66 118 123 10.1017/S0022149X00012694 1640085

[B41] Galan-Puchades MT Fuentes MV The Asian *Taenia* and the possibility of cysticercosis Korean J Parasitol 2000 38 1 7 10.3347/kjp.2000.38.1.1 10743352 PMC2721101

[B42] Galan-Puchades MT Fuentes MV *Taenia asiatica* intermediate hosts Lancet 2004 363 660 14987900 10.1016/S0140-6736(04)15607-9

[B43] Ito A Basic and applied immunology in cestode infections: from *Hymenolepis* to *Taenia* and *Echinococcus* Int J Parasitol 1997 27 1203 1211 10.1016/S0020-7519(97)00118-5 9394191

[B44] Ito A Fan PC Chung WC Suzuki M Cross protection against *Taenia taeniaeformis* in rats vaccinated with non-viable oncospheres of Asian *Taenia* or *T. saginata* J Helminthol 1994 68 83 85 10.1017/S0022149X00013535 8006391

[B45] Fan PC Chung WC Eom KS Ito A Vaccination trials against Taiwan *Taenia* eggs in pigs injected with frozen oncospheres of Taiwan *Taenia*, Korea *Taenia, T. saginata or T. solium* Parasitology 1997 114 Pt 6 541 544 9172425

[B46] Fan PC Chung WC Lin CY Wu CC Vaccination trials against *Taenia solium* eggs in pigs injected with frozen oncospheres of *T. solium* or *Taenia saginata asiatica* J Microbiol Immunol Infect 2003 36 96 100 12886959

[B47] Jeon HK Eom KS Immunoblot patterns of *Taenia asiatica* taeniasis Korean J Parasitol 2009 47 73 77 10.3347/kjp.2009.47.1.73 19290097 PMC2655338

[B48] Lee EG Bae YA Kim SH Diaz-Camacho SP Nawa Y Kong Y Serodiagnostic reliability of single-step enriched low-molecular weight proteins of *Taenia solium* metacestode of American and Asian isolates Trans R Soc Trop Med Hyg 2010 104 676 683 10.1016/j.trstmh.2010.07.011 20801471

[B49] Fan PC Chung WC Lin CY Wu CC Prevalence of taeniasis and enterobiasis among aboriginal children in mountainous areas of Taiwan Gaoxiong Yi Xue Ke Xue Za Zhi 1990 6 475 482 2213969

[B50] Fan PC Chung WC Lin CY Wu CC Studies of taeniasis in Taiwan. XIV. Current status of taeniasis among Yami aborigines on Lanyu Island, Taitung County, southeast Taiwan Gaoxiong Yi Xue Ke Xue Za Zhi 1992 8 266 271 1619702

[B51] Eom KS Jeon HK Rim HJ Geographical distribution of *Taenia asiatica* and related species Korean J Parasitol 2009 47 Suppl S115 124 10.3347/kjp.2009.47.S.S115 19885327 PMC2769216

[B52] Fan PC Chung WC Soh CT Kosman ML Eating habits of east Asian people and transmission of taeniasis Acta Trop 1992 50 305 315 10.1016/0001-706X(92)90065-6 1356301

[B53] Suroso T Margono SS Wandra T Ito A Challenges for control of taeniasis/cysticercosis in Indonesia Parasitol Int 2006 55 Suppl S161 165 16380290 10.1016/j.parint.2005.11.025

[B54] Wandra T Depary AA Sutisna P Margono SS Suroso T Okamoto M Craig PS Ito A Taeniasis and cysticercosis in Bali and North Sumatra, Indonesia Parasitol Int 2006 55 Suppl S155 160 16376140 10.1016/j.parint.2005.11.024

[B55] Eom KS Rim HJ Epidemiological understanding of *Taenia* tapeworm infections with special reference to *Taenia asiatica* in Korea Korean J Parasitol 2001 39 267 283 10.3347/kjp.2001.39.4.267 11775327 PMC2721212

[B56] Galan-Puchades MT Fuentes MV Neurcysticercosis, *Taenia asiatica* and Cheju Island in Korea Trends Parasitol 2001 17 469 470 11642258 10.1016/s1471-4922(01)02089-x

[B57] Eom KS Jeon HK Kong Y Hwang UW Yang Y Li X Xu L Feng Z Pawlowski ZS Rim HJ Identification of *Taenia asiatica* in China: molecular, morphological, and epidemiological analysis of a Luzhai isolate J Parasitol 2002 88 758 764 12197126 10.1645/0022-3395(2002)088[0758:IOTAIC]2.0.CO;2

[B58] Li T Craig PS Ito A Chen X Qiu D Qiu J Sato MO Wandra T Bradshaw H Li L Taeniasis/cysticercosis in a Tibetan population in Sichuan Province, China Acta Trop 2006 100 223 231 10.1016/j.actatropica.2006.11.003 17166477

[B59] Li T Chen X Yanagida T Wang H Long C Sako Y Okamoto M Wu Y Giraudoux P Raoul F Detection of human taeniases in Tibetan endemic areas, China Parasitology 2013 140 1602 1607 10.1017/S003118201300111X 23866973

[B60] Anantaphruti MT Yamasaki H Nakao M Waikagul J Watthanakulpanich D Nuamtanong S Maipanich W Pubampen S Sanguankiat S Muennoo C Sympatric occurrence of *Taenia solium*, *T. saginata*, and *T. asiatica*, Thailand Emerg Infect Dis 2007 13 1413 1416 10.3201/eid1309.061148 18252126 PMC2857269

[B61] Anantaphruti MT Okamoto M Yoonuan T Saguankiat S Kusolsuk T Sato M Sato MO Sako Y Waikagul J Ito A Molecular and serological survey on taeniasis and cysticercosis in Kanchanaburi Province, Thailand Parasitol Int 2010 59 326 330 10.1016/j.parint.2010.03.007 20380891

[B62] Anantaphruti MT Current status of taeniasis in Thailand Korean J Parasitol 2013 51 37 42 10.3347/kjp.2013.51.1.37 23467328 PMC3587747

[B63] Michelet L Dauga C Molecular evidence of host influences on the evolution and spread of human tapeworms Biol Rev Camb Philos Soc 2012 87 731 741 10.1111/j.1469-185X.2012.00217.x 22321512

[B64] Yamasaki H Current status and perspectives of cysticercosis and taeniasis in Japan Korean J Parasitol 2013 51 19 29 10.3347/kjp.2013.51.1.19 23467264 PMC3587745

[B65] Devleesschauwer B Aryal A Joshi DD Rijal S Sherchand JB Praet N Speybroeck N Duchateau L Vercruysse J Dorny P Epidemiology of *Taenia solium* in Nepal: is it influenced by the social characteristics of the population and the presence of *Taenia asiatica*? Trop Med Int Health 2012 17 1019 1022 10.1111/j.1365-3156.2012.03017.x 22643112

[B66] Yamasaki H Nakao M Sako Y Nakaya K Sato MO Mamuti W Okamoto M Ito A DNA differential diagnosis of human taeniid cestodes by base excision sequence scanning thymine-base reader analysis with mitochondrial genes J Clin Microbiol 2002 40 3818 3821 10.1128/JCM.40.10.3818-3821.2002 12354889 PMC130864

[B67] Yamasaki H Allan JC Sato MO Nakao M Sako Y Nakaya K Qiu D Mamuti W Craig PS Ito A DNA differential diagnosis of taeniasis and cysticercosis by multiplex PCR J Clin Microbiol 2004 42 548 553 10.1128/JCM.42.2.548-553.2004 14766815 PMC344500

[B68] Jeon HK Chai JY Kong Y Waikagul J Insisiengmay B Rim HJ Eom KS Differential diagnosis of *Taenia asiatica* using multiplex PCR Exp Parasitol 2009 121 151 156 10.1016/j.exppara.2008.10.014 19017531

[B69] Gonzalez LM Bailo B Ferrer E Garcia MD Harrison LJ Parkhouse MR McManus DP Garate T Characterization of the *Taenia* spp HDP2 sequence and development of a novel PCR-based assay for discrimination of *Taenia saginata* from *Taenia asiatica* Parasit Vectors 2010 3 51 10.1186/1756-3305-3-51 20540755 PMC2906438

[B70] Jeon HK Kim KH Chai JY Yang HJ Rim HJ Eom KS Sympatric distribution of three human *Taenia* tapeworms collected between 1935 and 2005 in Korea Korean J Parasitol 2008 46 235 241 10.3347/kjp.2008.46.4.235 19127329 PMC2612608

[B71] Chai JY Human taeniasis in the Republic of Korea: hidden or gone? Korean J Parasitol 2013 51 9 17 10.3347/kjp.2013.51.1.9 23467688 PMC3587755

[B72] Jeon HK Kim KH Eom KS Molecular identification of *Taenia* specimens after long-term preservation in formalin Parasitol Int 2011 60 203 205 10.1016/j.parint.2010.12.001 21163367

[B73] Wandra T Sutisna P Dharmawan NS Margono SS Sudewi R Suroso T Craig PS Ito A High prevalence of *Taenia saginata* taeniasis and status of *Taenia solium* cysticercosis in Bali, Indonesia, 2002–2004 Trans R Soc Trop Med Hyg 2006 100 346 353 10.1016/j.trstmh.2005.06.031 16199069

[B74] Conlan JV Sripa B Attwood S Newton PN A review of parasitic zoonoses in a changing Southeast Asia Vet Parasitol 2011 182 22 40 10.1016/j.vetpar.2011.07.013 21846580

[B75] Wandra T Ito A Swastika K Dharmawan NS Sako Y Okamoto M Taeniases and cysticercosis in Indonesia: past and present situations Parasitology 2013 140 1608 1616 10.1017/S0031182013000863 23965293

[B76] Wang ZR Bao HE Identification of *Taenia saginata* by mtCO I in four areas of Yunnan and Guizhou provinces Zhongguo Ji Sheng Chong Xue Yu Ji Sheng Chong Bing Za Zhi 2003 21 20 23 12884584

[B77] Liu AB Yang YM Analysis of the mitochondrial DNA-gene encoding ribosomal RNA small subunit gene sequence of *Taenia* cestode from Baoshan and Puer areas in Yunnan Province Chinese Journal of Infectious Diseases 2011 29 236 238

[B78] Yan H Lou Z Li L Ni X Guo A Li H Zheng Y Dyachenko V Jia W The nuclear 18S ribosomal RNA gene as a source of phylogenetic information in the genus *Taenia* Parasitol Res 2013 112 1343 1347 10.1007/s00436-012-3199-9 23183704

[B79] Yang YC Ou-Yang Y Su AR Wan XL Li SL Analysis of COX1 sequences of *Taenia* isolates from four areas of Guangxi Zhongguo Xue Xi Chong Bing Fang Zhi Za Zhi 2012 24 307 310 23012955

[B80] Chen Y Bao HE Li JF Lang SY Qiu XL Huang J Wu YM Zhang CY Epidemiological investigation of *Taenia saginata asiatica* in Duyun, Guizhou and detection of amino acids and elements of adult worms Zhongguo Ji Sheng Chong Xue Yu Ji Sheng Chong Bing Za Zhi 2003 21 311 313 15108532

[B81] Mou R Bao HE Qiu XL Chen Y Lang SY Huang J Li JH Zhu WJ Zhang K Ling-Hu Y Morphological observation on the adult worms of *Taenia saginata* in western China Zhongguo Ji Sheng Chong Xue Yu Ji Sheng Chong Bing Za Zhi 2007 25 32 35 17639696

[B82] Nkouawa A Sako Y Li T Chen X Nakao M Yanagida T Okamoto M Giraudoux P Raoul F Nakaya K A loop-mediated isothermal amplification method for a differential identification of *Taenia* tapeworms from human: application to a field survey Parasitol Int 2012 61 723 725 10.1016/j.parint.2012.06.001 22698671

[B83] Van De N Molecular identification of *Taenia asiatica* isolated from a patient in Ha Tay province of Vietnam Journal of Medical and Pharmaceutical Information 2003 10 28 32

[B84] Somers R Dorny P Geysen D Nguyen LA Thach DC Vercruysse J Nguyen VK Human tapeworms in north Vietnam Trans R Soc Trop Med Hyg 2007 101 275 277 10.1016/j.trstmh.2006.04.007 16806333

[B85] Ohnishi K Sakamoto N Kobayashi K Iwabuchi S Nakamura-Uchiyama F Therapeutic effect of praziquantel against taeniasis *asiatica* Int J Infect Dis 2013 17 e656 657 10.1016/j.ijid.2013.02.028 23618773

[B86] Amin MR Rabbi SF Zaman MF Rahman MK Pork tapeworm (*Taenia saginata asiatica*) infection in rural Bangladesh J Med 2009 10 135 138

[B87] Jeon HK Yong TS Sohn WM Chai JY Hong SJ Han ET Jeong HG Chhakda T Sinuon M Socheat D Eom KS Molecular identification of *Taenia* tapeworms by Cox1 gene in Koh Kong, Cambodia Korean J Parasitol 2011 49 195 197 10.3347/kjp.2011.49.2.195 21738280 PMC3121081

[B88] Gasser RB Zhu X McManus DP NADH dehydrogenase subunit 1 and cytochrome c oxidase subunit I sequences compared for members of the genus *Taenia* (Cestoda) Int J Parasitol 1999 29 1965 1970 10.1016/S0020-7519(99)00153-8 10961852

[B89] Yamasaki H Nakao M Sako Y Nakaya K Ito A Molecular identification of *Taenia solium* cysticercus genotype in the histopathological specimens Southeast Asian J Trop Med Public Health 2005 36 Suppl 4 131 134 16438197

[B90] Le T De N Doanh N Nga N Molecular identification and phylogenetic analysis of human parasitic *Taenia* sp isolated in Vietnam Journal of Malaria and Parasite Diseases Control 2003 0 65 73

[B91] Gonzalez LM Ramiro R Garcia L Parkhouse RM McManus DP Garate T Genetic variability of the 18 kDa/HP6 protective antigen in *Taenia saginata* and *Taenia asiatica*: implications for vaccine development Mol Biochem Parasitol 2011 176 131 134 10.1016/j.molbiopara.2010.12.014 21232558

[B92] Morakote N Wijit A Uparanukraw P Further search for *Taenia saginata asiatica* in Chiang Mai, Thailand Ann Trop Med Parasitol 2000 94 521 524 10983566 10.1080/00034983.2000.11813572

[B93] Gonzalez LM Montero E Morakote N Puente S Diaz De Tuesta JL, Serra T, Lopez-Velez R, McManus DP, Harrison LJ, Parkhouse RM, Garate T: **Differential diagnosis of *****Taenia saginata *****and *****Taenia saginata asiatica *****taeniasis through PCR** Diagn Microbiol Infect Dis 2004 49 183 188 10.1016/j.diagmicrobio.2004.03.013 15246508

[B94] Zarlenga DS George M *Taenia crassiceps*: cloning and mapping of mitochondrial DNA and its application to the phenetic analysis of a new species of *Taenia* from Southeast Asia Exp Parasitol 1995 81 604 607 10.1006/expr.1995.1155 8543003

[B95] Michelet L Carod JF Rakontondrazaka M Ma L Gay F Dauga C The pig tapeworm *Taenia solium*, the cause of cysticercosis: Biogeographic (temporal and spacial) origins in Madagascar Mol Phylogenet Evol 2010 55 744 750 10.1016/j.ympev.2010.01.008 20093191

[B96] Anantaphruti MT Thaenkham U Watthanakulpanich D Phuphisut O Maipanich W Yoonuan T Nuamtanong S Pubampen S Sanguankiat S Genetic diversity of *Taenia asiatica* from Thailand and other geographical locations as revealed by cytochrome c oxidase subunit 1 sequences Korean J Parasitol 2013 51 55 59 10.3347/kjp.2013.51.1.55 23467439 PMC3587750

[B97] Anantaphruti M Thaenkham U Kusolsuk T Maipanich W Saguankiat S Pubampen S Phuphisut O Genetic variation and population genetics of *Taenia saginata* in North and Northeast Thailand in relation to *Taenia asiatica* J Parasitol Res 2013 2013 310605 23864933 10.1155/2013/310605 PMC3707265

[B98] Galan-Puchades MT Fuentes MV *Taenia asiatica*: the most neglected human *Taenia* and the possibility of cysticercosis Korean J Parasitol 2013 51 51 54 10.3347/kjp.2013.51.1.51 23467406 PMC3587749

[B99] Conlan JV Vongxay K Fenwick S Blacksell SD Thompson RC Does interspecific competition have a moderating effect on *Taenia solium* transmission dynamics in Southeast Asia? Trends Parasitol 2009 25 398 403 10.1016/j.pt.2009.06.005 19717341

[B100] Flisser A State of the art of *Taenia solium* as compared to *Taenia asiatica* Korean J Parasitol 2013 51 43 49 10.3347/kjp.2013.51.1.43 23467388 PMC3587748

[B101] Dorny P Praet N *Taenia saginata* in Europe Vet Parasitol 2007 149 22 24 10.1016/j.vetpar.2007.07.004 17706360

[B102] Ito A Nakao M Wandra T Suroso T Okamoto M Yamasaki H Sako Y Nakaya K Taeniasis and cysticercosis in Asia and the Pacific: present state of knowledge and perspectives Southeast Asian J Trop Med Public Health 2005 36 Suppl 4 123 130 16438196

